# Pulse-Cereal Blend Extrusion for Improving the Antioxidant Properties of a Gluten-Free Flour

**DOI:** 10.3390/molecules26185578

**Published:** 2021-09-14

**Authors:** Daniel Rico, Ana Belén Cano, Ana Belén Martín-Diana

**Affiliations:** Subdirection of Research and Technology, Agro-Technological Institute of Castilla y León, Consejería de Agricultura y Ganadería, Finca de Zamadueñas, Ctra. Burgos km. 119, 47171 Valladolid, Spain; cannunan@itacyl.es

**Keywords:** extrusion, lentil, corn, rice, antioxidant, glycemic index, SEM

## Abstract

Extrusion is an interesting technological tool that facilitates pulse formulation into flour mixtures, with tailored fibre content, total antioxidant capacity (TAC) and glycemic index (GI) among other components in final formulas. The gluten-free (GF) market has significantly grown during the last years. GF products have evolved from specialty health foods to products targeted to the general population and not only associated to celiac consumers. This study evaluates how temperature, cereal base (rice/corn) and pulse concentration affect extruded flour properties and which conditions are more efficient to develop a gluten-free flour with high TAC and low GI. Additionally, it evaluated the effect of this optimal formula after the baking process. The results showed an increase of total phenol (TP) and antioxidant activity with extrusion, with a temperature-dependent effect (130 °C ≥ 120 °C ≥ 110 °C), which may imply an enhanced bioaccessibility of phenolics compounds after extraction. Extrusion increased GI in comparison to native flour; however, a dough temperature of 130 °C resulted in a significantly (*p* ≤ 0.05) lower GI than that observed for 110–120 °C doughs, probably associated to the pastification that occurred at higher temperatures, which would decrease the degree of gelatinization of the starches and therefore a significant (*p* ≤ 0.05) GI reduction. Corn-lentil flour showed higher antioxidant properties and lower GI index in comparison with rice-lentil blends. The formulation of the optimal blend flour into a baked product (muffin) resulted in a significant loss of antioxidant properties, with the exception of the reducing power (FRAP), although the final antioxidant values of the baked product were in the range of the original native flour blend before any process.

## 1. Introduction

Celiac disease (CD) is an autoimmune illness which is highly determined genetically, but with an important influence of other factors, mainly diet, which are still of controversy. The incidence of this disease is increasing in recent years [1], and it has been proposed an iceberg model with the majority of the cases undiagnosed [2]; this suggests a deeper complexity of the celiac disease, with milder levels of symptomatology, and resulting in a wider percentage of the population potentially affected by gluten consumption.

Strict adherence to a gluten-free diet (GFD) is the primary frontline treatment for celiac disease. Following a GFD is not easy, due to food-related social issues, and unintended gluten contamination of many foods. Furthermore, following a gluten-free diet may increase the possibility of suffering nutritional deficiencies which can cause malnutrition. Between 20% to 40% of celiac patients had nutritional unbalance, mainly related to iron and vitamin B_12_ deficiencies [3]. In addition, the cost of gluten-free (GF) products can easily triplicate that of the gluten-containing counterparts [4,5,6].

In the case of a GFD, it requires the complete exclusion of gluten, a protein complex present in wheat, barley and rye. According to FAO [7], an adult’s diet should provide a daily consumption of 0.59 g of proteins of high biological value per kg weight and day. In the case of vegetable proteins, this contribution should be higher due to their lower digestibility, being recommended a contribution between 0.9 and 1.2 g kg^−1^ day^−1^ [8].

New flour alternatives for developing nourishing gluten-free products that mimic gluten-containing counterparts are required. Moreover, the lack of functional properties of GF flours compels the incorporation of hydrocolloids and concentrates of animal protein to form a foamy structure in gluten-free doughs. Xanthan gum is common in GF formulations and, although it does not raise any safety concern, its consumption can cause abdominal discomfort in some susceptible individuals [9]. Gluten-free baked products are mostly based on flour from rice or corn with low content and biologically poor protein [10]. For this reason, combination of these cereals with novel nutrient-rich matrices is essential.

Pulses are protein-rich alternatives to cereals as staple food, being a good source of carbohydrates, soluble fibre, calcium, phosphorus, potassium, magnesium, iron and B vitamins [11,12,13]. The amino acid profile of legumes, limited in methionine, is complementary to cereals, limited in lysine, and it is recommended a combined consumption in order to increase the quality of the total dietary protein [14].

Lentil (*Lens culinaris* Medik.) is native to Western Asia and North America; it is one of the earliest domesticated crops and part of the human diet since ancient times. The nutritional characteristics of lentils have been associated with cholesterol- and lipid-lowering effects in humans, along with reducing the incidence of colon cancer and type-2 diabetes [15,16]. Moreover, the consumption has been associated to improved blood pressure [17], anti-inflammatory properties [18], and even a satiety effect [19].

Extrusion is a growing technology for the food industry, which processes and markets an important number of extrusion-derived products, from pasta to baby foods. Cooking extrusion allows working with a variety of raw materials and their mixtures at a certain range of moisture and temperature conditions. Extrusion of cereal flours cooked into expanded snack products is well documented [20,21,22,23,24]. In this sense the extrusion technology represents an important advantage for the industry, since it allows to transform flour to obtain foods rich in nutrients and low in fat content, thus enhancing healthier and more sustainable food [21].

Flour during the extrusion process is subjected to high temperature and high shear at relatively low levels of moisture content, which can modify the functionality of flour mixtures such as gelatinization and degradation of starch, solubilization of dietary fibre, and protein aggregation. It can also help to reduce the levels of some anti-nutrients contained in pulses, such as tannins, phytic acid, and trypsin inhibitors [25,26,27]. In addition, extrusion cooking is able to increase the digestibility of starch and proteins, increase the bioavailability of bioactive and nutraceutical compounds and reduce the glycemic-index (GI) [27]. Although low GI is not a requirement for celiac population, there is increasing evidence that a low-glycaemic-index diet can be beneficial, improving metabolic control of hyperglycaemia and hyperlipidaemia in diabetic patients, as well as in healthy population [28,29,30,31,32]. This is of special importance in the case of celiac since many gluten-free products are formulated with refined starch matrices. In this sense, legumes were identified as low GI foods more than 20 years ago [31,32].

To introduce pulse flours in final products requires previous process optimization, such as feeding mixture, moisture content, screw speed, temperature of the barrel and compression ratio to obtain certain functional and bioactive properties. Pulses such as chickpeas, beans, peas or cowpeas, have been well studied in extrusion [33,34,35]; however, fewer studies have been conducted on lentil or lentil blend flour extrusion.

Developing healthy GF products is an important challenge for the industry due to a heavy reliance on refined flours. For this reason, the main objective was to obtain a nutrient-rich flour with improved antioxidant properties and glycemic index suitable for new GF product formulation through the application of extrusion technology and optimisation cereal used as blend formulation, processing temperature and pulse concentration.

## 2. Results and Discussion

### 2.1. Optimization of Extrusion Temperature and Blend Mixture (Step 1)

Proximal composition was evaluated for native flours (corn, rice and lentil) and corn, rice and blend flours (50:50) extruded at different exit temperatures (110, 120, and 130 °C, Table 1). Extrusion produced a significant effect on several nutritional parameters of the flours. Native flours (NF) showed ash content values from 0.53 to 2.17 g 100 g^−1^, and the highest values were observed for lentil flour. Ash lentil values agreed with those reported by Espeso Blanco (2.52 g 100 g^−1^) [35], and Ghumman et al. (2.12 to 3.54 g 100 g^−1^) [36]. Corn and rice ash values were also similar to results published by Awuchi et al. [37]. The extrusion produced a reduction in ash content in the case of corn and lentil:corn blend flours, and no significant (*p* > 0.05) reduction was observed in rice and lentil:rice flours. Ash reduction has been associated to the interaction of minerals with fibre and protein [38] or to lixiviation and vaporization processes during extrusion [35].

The ash reduction observed in rice, and lentil blends with rice or corn was significant at 110 and 120 °C, and no reduction was observed when exit temperature reached 130 °C. Corn flour extruded at 110 °C had lower ash content than flours extruded at higher temperatures (120 and 130 °C). Contrary to this, reduction of ash content with increasing extrusion temperature has been previously observed [37].

Fat content for NF ranged from 0.86 in the case of rice and corn to 1.42 g 100 g^−1^ in lentil; these values agreed with those reported by other authors [35,39,40,41,42]. The amylose component of gelatinised starch is known to interact with lipids [42]. It was observed a general reduction in fat content, which would respond to the interaction between lipids and amylose during extrusion and would reduce the extractability of fat. Similar findings were reported previously [35,43,44]. The higher decrease in fat content in the mixture lentil:corn (elcf) flour as compared with lentil:rice (elrf) flour may correspond to the higher amylose:amylopectin ratio of corn [45].

The flour moisture content ranged from 9.75 g 100 g^−1^ to 13.64 g 100 g^−1^, being corn and lentil flours where highest and lowest moisture values were observed, respectively. The moisture content decreased significantly (*p* ≤ 0.05) after extrusion process in all the flours, being the temperature a significant factor; those flours extruded at a higher final temperature showed significantly (*p* ≤ 0.05) lower moisture content. Blend flours showed values from 7.15 to 11.40 g 100 g^−1^, with the lowest moisture values in rice mixture flours at high temperatures.

The protein content of lentil:corn and lentil:rice mixtures, according to protein content of flours and a 50:50 proportion, will be 15.17 and 16.05, respectively. Significant changes in protein content were not observed after the extrusion process. The extrusion increases hydrophobic interaction of proteins with other compounds such as starch and lipids. Denaturation of proteins at high temperature during extrusion cooking may improve protein digestibility, associated to protein unfold, hydrolysis, and cross-linking with other ingredients, although increased protein content may alter starch gelatinization through water competition [46,47].

Carbohydrates suffered modification after extrusion (Table 1); a slight increment was observed after the process in all the samples. In addition, temperature had a significant effect on this parameter, with increasing carbohydrate content in those samples treated at higher temperature. This increment may be related to a higher proportion of lipids bound to starch, which may be retained in the matrix after fat extraction [48].

The extrusion process produced a reduction of fibre content in corn flour and an increase in rice, lentil:corn and lentil:rice flours; this effect was associated to the temperature and blend with lentil flour, as blend flours extruded at high temperature (130 °C) showed a significant reduction in fibre, after increases at 110 and 120 °C. The results agree with findings reported by Frias et al. [49], who reported a decrease in TDF from 7% to 16% in *Pisum sativum* L. flour after extrusion at 129–142 °C. Jan et al. [50] also explained the reduction of fibre may be due to hydrolysis during extrusion process, which will enhance a precipitation of fibre molecules, reducing the observed values. This reduction in fibre has been explained with a redistribution of insoluble to soluble dietary fibre during the extrusion process [23,51].

Phytic acid suffered a significant reduction in blend flours during the extrusion; however, the reduction was not affected by temperature, flours extruded to 110 °C showed the same reduction in phytic acid than flours extruded at 130 °C, probably associated to the main mechanism involved in reduction phytic acid is the hydrolysis and the action of phytase with optimum activity at 48 to 55 °C [26,52,53]. Corn and rice showed low value of phytic acid and the extrusion did not produce a significant decrease on phytic acid. However, the values of phytic acid in native rice were significantly lower than values reported by Albarracin et al. [54] who found values of 740.09 mg 100 g^−1^ probably differences are related with variety of rice used. The same results were observed in corn where values were 100 time lower than values reported by Coulibaly et al. [55].

Colorimetric and image analysis were analysed in order to evaluate the effect of extrusion temperature. Changes in luminosity (Table 2) were observed in all the extruded flours; samples extruded at 130 °C showed significant (*p* ≤ 0.05) reductions in L* values, regardless the flour or blend used. This reduction in luminosity was higher in rice flour (84.06 NF to 32.64 extruded flour at 130 °C in a scale 0 to 100) than in corn (72.52 NF to 52.24 extruded flour at 130 °C). This decrease in luminosity and significance in b* values may be attributed to a sufficient presence of reducing sugars in extruded flour for Maillard reaction [56,57]. In the case of rice flours (rf, erf), extrusion showed a decrease in L* and increase in a* and b* values, results similar to those observed by Lei et al. [58]. Ilo and Berghofer [59] observed that luminosity and a* values were parameters highly affected by extrusion and may be of utility to predict degree of cooking on the final product. Combination of lentil flour with rice resulted in increased a* values; this was also observed by Chakraborty et al. [60], who associated this to increased amounts of amino acids in the extruded mixture. The b* values were in the range of positive values (yellowness) and showed a different behaviour in the case of corn and rice flours, decreasing after extrusion in the first case, and increasing in the case of rice. The decrease in the case of corn of b* values may be explained as carotenoid loss after extrusion [61].

Hue and chroma also changed significantly (*p* < 0.05); Hue increased and chroma was reduced. Colour changes not only play an important role in product acceptance by consumers [62], but also indicate molecular and chemical changes, such as non-enzymatic browning, as a result of the extrusion process.

**Figure 1:**
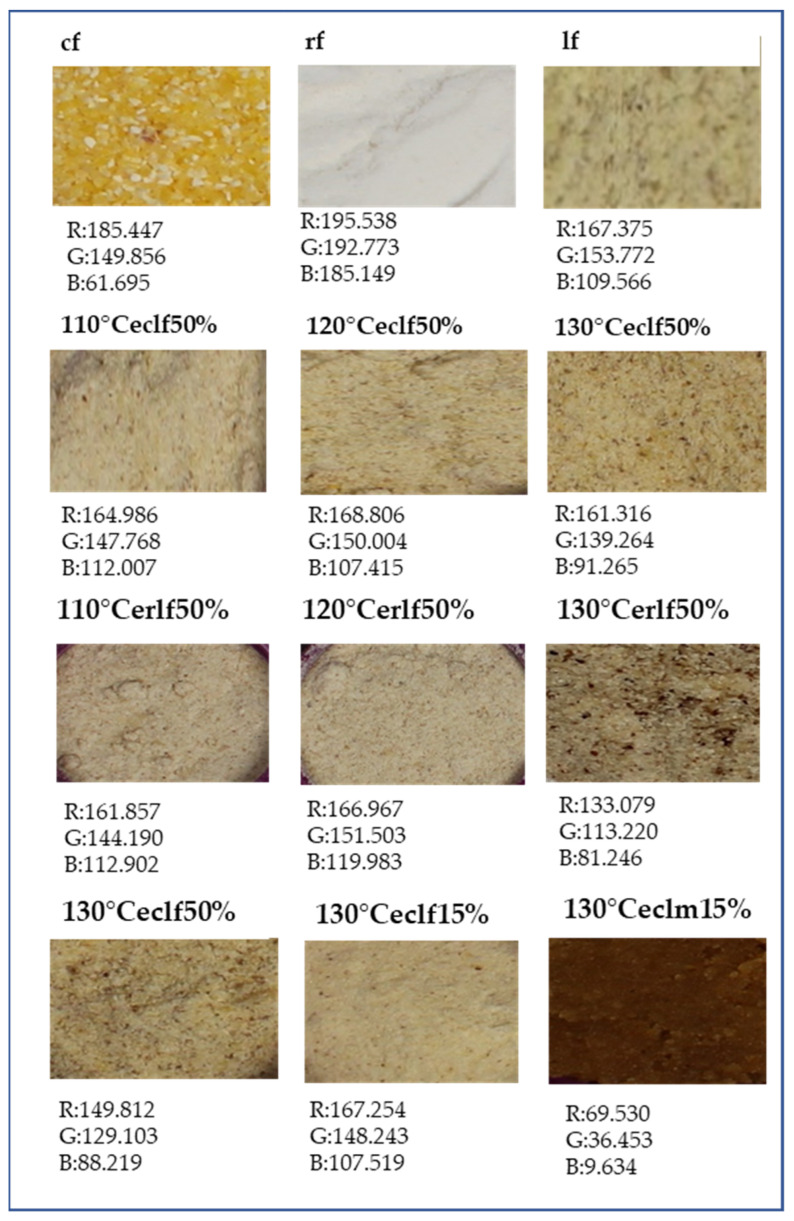
Image analysis (R: red; G: green; B; brown) of native and extruded flours. Abbreviations: cf: corn flour; rf: rice flour; lf: lentil flour; ecf: extruded corn flour; erf: extruded rice flour; elcf: extruded lentil (50% or 15%)-corn flour; elrf: extruded lentil (50% or 15%)-rice flour; elcm: baked product with extruded lentil-corn flour.

Colour analyses were completed with the evaluation of RGB (red, green and blue) parameters from product images (Figure 1). The image analysis showed a reduction in R values probably due to non-enzymatic browning reactions (Maillard and caramelization) during extrusion. This effect was more significative when the addition of lentil increased and after the baked process.

**Figure 2:**
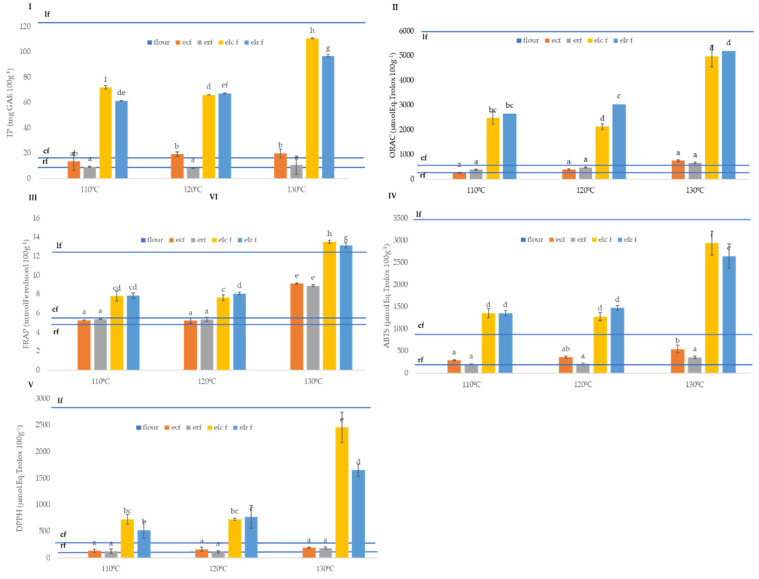
Total phenol (TP, mg GAE 100 g^−1^ dry matter, (**I**)), total antioxidant activity as ORAC, ABTS•+, DPPH ((**II**–**V**), µmol Eq. Trolox 100 g^−1^ dry matter), and FRAP (mmol Fe^2+^ 100 g^−1^ dry matter) of native and extruded flours. Abbreviations: cf: corn flour; rf: rice flour; lf: lentil flour; ecf: extruded corn flour; erf: extruded rice flour; elcf: extruded lentil (50%)-corn flour; elrf: extruded lentil (50%)-rice flour. Different small letters indicate differences (*p* ≤ 0.05) between samples.

Total phenol (TP) was evaluated in native flours and extruded flours using the Folin Ciocalteu reagent method and the results were expressed as mg of GAE 100 g^−1^ (Figure 2I). The analyses showed significant differences between NF in TP content, with values ranging from 10.38 mg GAE 100 g^−1^ in rice flours, 29.48 mg GAE 100 g^−1^ in corn flour, and 222 mg GAE 100 g^−1^ in lentil flour. Corn flour showed TP values slightly lower than those reported by Zhang et al., [63] who analysed more than eight different corn varieties and reported values between 38.00 and 57.04 mg GAE 100 g^−1^. The differences on TPs may be associated with cultivar, agronomic practice, and extraction procedure since the method also measures other reducing substances such as flavonoids, proteins, sugars, etc. which can interfere in the analytical method and produce phenolic content overestimation [64].

TP in rice flours showed similar values to those reported by Thanuja & Parimalavalli [64], who evaluated different rice varieties, finding TP values from 20 to 26 mg GAE 100 g^−1^ in white rice cultivars. In the case of lentil flours, the values were significantly higher than those observed by Kalogeropoulos et al. [65], who found TP values from 25.9 mg GAE 100 g^−1^, and lower than those obtained by Morales et al. [66], who found values of 1413 mg Ferulic acid Eq. 100 g^−1^. The important differences observed in our results and those reported by other authors, up to one order of magnitude, may be due to cultivar variety or extraction procedure [64].

Results showed than extrusion did not produce a significant (*p* ≤ 0.05) reduction in TP content, as compared with native flours. Conversely, increasing temperatures (130 °C) resulted in samples with higher amount of TP than those extruded at lower temperatures (110–120 °C), an effect observed only on the blend lentil:cereal mixtures. This increase of the phenolic content in blend samples extruded at higher temperature (130 °C) may respond to the important contribution of bound phenolics by lentil [67] and the structure modification induced by cell rupture at higher temperature, producing higher cell wall porosity, and improving the diffusion of solvent during extraction, enhancing the availability of these compounds [66,68,69]. Another explanation may be the hydrolysis of bound polyphenols associated to fibre and/or proteins moieties, changing from non-extractable to extractable polyphenols, as suggested by Korus, Gumul, and Czechowska [70]. In addition, this increment in TPs can respond the neogeneration of Maillard reaction products (MRP) which react as reducing agents with Folin Ciocalteau reagent in the TP assay and are more abundant in pulses flours due to the high content in amino acids related with Maillard reaction [71]. However, controversy information has been published in this sense; Alonso et al., [72] reported that bean flour extrusion at 150–155 °C did not produce any significant change in total phenolic content and antioxidant activity. Other authors suggest that after thermal treatments such as extrusion, there is a reduction of TP associated with phenolic thermolability and decarboxylation effect due to the temperature, which may promote polymerisation of phenols, reducing their extractability [73]. Yeo and Shahidi [74] suggested that thermal treatment reduced soluble and insoluble phenolic fractions in lentil due to the formation of irreversible covalent bonds between free phenolic compounds and proteins.

Total antioxidant capacity was also evaluated (Figure 2II–V, Table 3) in all the flours through the use of different parameters (ORAC, ABTS•+, DPPH, FRAP, Q-DPPH and Q-ABTS•+) using extractive and direct methods. The results showed that extrusion did not produce a significant (*p* ≤ 0.05) reduction on the antioxidant activity of the extruded flours compared to the native flours, with the exception of the antioxidant capacity against DPPH and ABTS•+•+ radicals, in which corn and lentil blend flours suffered a decrease when treated at 110 and 120 °C, as compared to the native flour. The use of higher extrusion temperature (130 °C) produced significant (*p* < 0.05) increases of antioxidant activity in blend flours, as measured with the different antioxidant capacity methods. The observed increases in activity of blend flours extruded at 130 °C was between 50% and 100%. In the case of corn and rice flours, this effect was not observed except in the case of FRAP results, where a significant increase in corn and rice extruded flours is observed at 130 °C. A significant increase of the antioxidant activity, as evaluated through the DPPH and FRAP methods, after extrusion and with a positive effect of increasing extrusion temperatures has been also observed previously in lentil formulations [75]. Similar results have also been reported for other legume-cereal blends [76].

The significant correlation (Supplementary Figure S3I) observed among total phenols (TP) and the different antioxidant markers (ORAC, FRAP, ABTS•+•+ and DPPH) would explain the contribution of TP to this increment in antioxidant activity within the Maillard derivates such as melanoids produced at high temperatures observed [77]. Since at higher temperatures, where higher molecular weight (MW) Maillard compounds are produced such as higher antioxidant compounds and antioxidant activity, those produced at 130 °C will have higher MW and also higher antioxidant capacity [78,79].

The results showed that rice flours after extrusion significantly reduced (*p* ≤ 0.05) its antioxidant capacity compared with corn flours, which may demonstrate that the antioxidants in rice flours are more vulnerable and thermally sensitive than in corn flours [80].

Methods to evaluate antioxidant capacity of solid samples without extraction were also used. Direct methods give an estimation of the antioxidant capacity of the insoluble fraction of antioxidants, or compounds bound to the insoluble matrix, which in the case of cereal and legume flours, fruit and vegetables can be significantly higher than the soluble fraction [81]. This procedure helps to overcome the drawbacks of extraction-dependent classical methods leading to underestimation of the total antioxidant capacity (TAC) of foods.

Antioxidant methods on solid samples (Q-DPPH and Q-ABTS•+) showed higher antioxidant activity than the methods on extracts, as expected (Table 3). Q-DPPH showed that rice native flour had the lowest antioxidant activity, followed by corn and lentil native flours (65.51 ± 8.16, 489.40 ± 153.80, 2100.23 ± 237.96 µmol Eq. Trolox 100 g^−1^, respectively). These results correlate well with the fibre content found before extrusion (1.65 ± 0.00, 4.05 ± 0.49, 15.05 ± 2.19, for rice, corn and lentil native flours, respectively). The extrusion process at 110 and 120 °C resulted in significant (*p* ≤ 0.05) reductions of scavenging activity in all the flours; conversely, antioxidant capacity increased in the case of flour extruded at higher temperature (130 °C). This lower reduction in antioxidant activity can be due to the increment of hydrolysis of fibre due to extrusion process which enhance the transformation of bound phenolic compounds to free phenolic compounds. Q-DPPH and Q-ABTS•+ initial reduction after extrusion at 110 and 120 °C may be associated with a pastification effect [82] which may result in a reduced availability of antioxidant compounds at the surface of the matrix and lower antioxidant observed on the solid samples. The hydrolysis of the fibre fraction during extrusion [83], which may increase fibre surface interaction with radicals during the direct antioxidant assays on solid samples, may explain that at higher temperature (130 °C) the antioxidant capacity of the samples was significantly increased, reaching values over those initially found on native flours.

The results of the antioxidant activity obtained by the above-explained antioxidant methods were integrated by calculating the Relative Antioxidant Capacity Index (RACI). The RACI allows the comparison of antioxidant capacity derived from ABTS•+, DPPH, ORAC, FRAP, including the TP. RACI values (Supplementary Table S1) showed than lentil had a significant (*p* ≤ 0.05) higher values than corn or rice flours. Flours obtained at higher extrusion temperatures had higher RACI values than those extruded at lower temperatures and a significant effect of lentil concentration also was observed being flour with 50% where higher values were observed.

**Figure 3:**
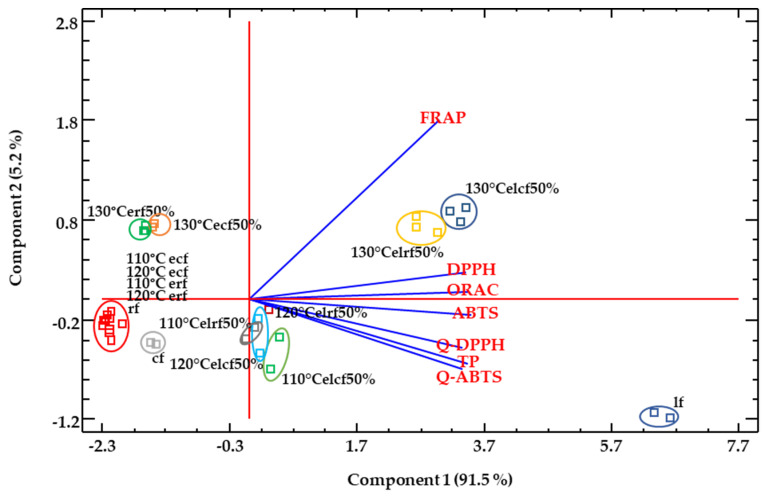
PCA analysis for native flours and extruded flours at different conditions. Abbreviations: cf: corn flour; rf: rice flour; lf: lentil flour; ecf: extruded corn flour; erf: extruded rice flour; elcf: extruded lentil (50%)-corn flour; elrf: extruded lentil (50%)-rice flour.

A principal components analysis (Figure 3) was performed in order to obtain an overview of the similarities and differences between all the studied samples, as well as to investigate the relationship between the different methods used for evaluating the antioxidant activity. The different antioxidant capacity methods showed high correlation values amongst them (Supplementary Figure S1). The first two principal components, PC_1_ and PC_2_, explain 91.49% and 5.15% of the total variance of the data set, respectively (Figure 3). The analysis showed that the extrusion produced a separation of flours associated with the temperature; flours extruded at 130 °C had a higher reducing power than those extruded at 110 °C and or 120 °C. Conversely, the antioxidant markers (TP, ORAC, ABTS•+, DPPH, Q-ABTS•+, Q-DPPH) were well correlated with incorporation of lentil flour in the formulation, resulting in higher antioxidant values in samples containing lentil flour. Native lentil flours were clearly differentiated from rest of the samples, mainly due to differences in Q-methods and TPs. This behaviour can respond to the low solubility of lentil antioxidants previous to extrusion the process, which may be released after extrusion. Other authors have reported that relatively mild conditions in the extrusion processing (<140 °C) limit the Maillard reaction products occurrence, compounds that show significant antioxidant capacity [79]. In any case, as observed from colour results, Maillard reaction products may have contributed to the antioxidant capacity increase with increasing temperature, as well as the bound phenolic fraction released during extrusion [80].

**Figure 4:**
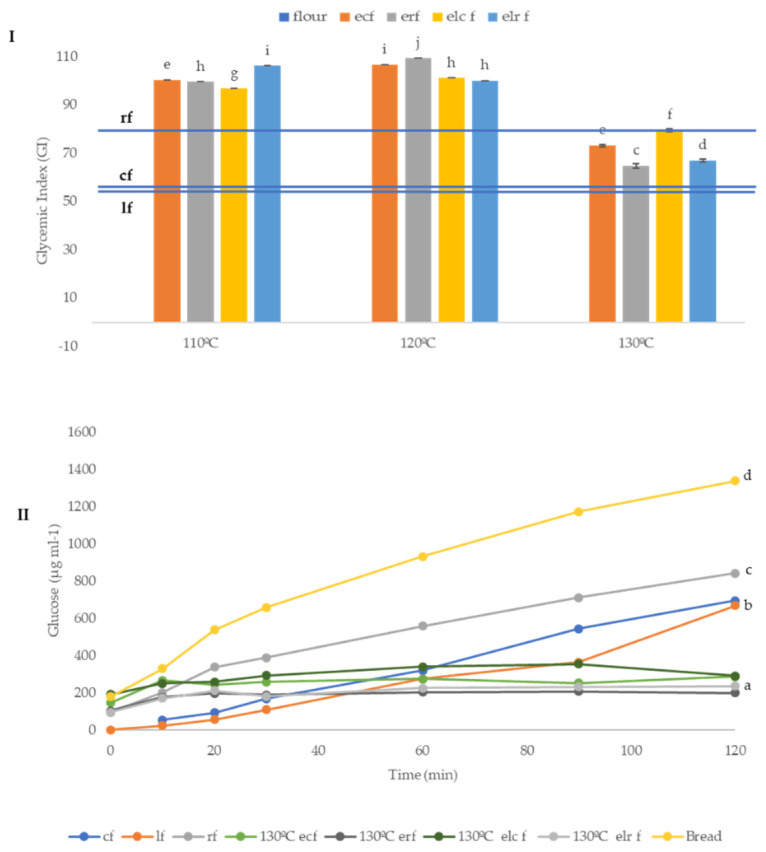
Glycemic index (GI, (**I**)) and glucose kinetic consumption (µg mL^−1^) (**II**) of native and extruded flours. Abbreviations: cf: corn flour; rf: rice flour; lf: lentil flour; ecf: extruded corn flour; erf: extruded rice flour; elcf: extruded lentil (50%)-corn flour; elrf: extruded lentil (50%)-rice flour. Different small letters indicate significant differences (*p* ≤ 0.05) between samples.

The glycemic index (GI) was evaluated in native and extruded flours. Extrusion (Figure 4I) produced an increment in the glycemic index of the different samples extruded at 110 and 120 °C, with a reduction observed when samples were extruded at 130 °C. The observed effect at 110 and 120 °C may be due to the gelatinization process under extrusion cooking conditions. Moreover, extrusion affects the particle size, producing a disruption of cell wall components [69], which may facilitate accessibility to starch by amylases. Conversely, the observed reduced GI of samples extruded at 130 °C could respond to a pastification process, favoured at higher temperatures, and resulting in the formation of a shell layer, which limits starch gelatinization and access by digestion enzymes [82].

The release of glucose from starch during in vitro digestion kinetics was slowed down when the samples were extruded at 130 °C (Figure 4II), probably due to the presence of resistant starch, which is related to lower GI values [84]. When the kinetic glucose was monitored, it was observed that, during the first 20 min, the glucose release was lower in non-extruded flours, especially lentil and corn; however, after 25 min, extruded flours at 130 °C had lower glucose release than native flours or flours extruded al lower temperatures 110 or 120 °C (data not shown).

**Figure 5:**
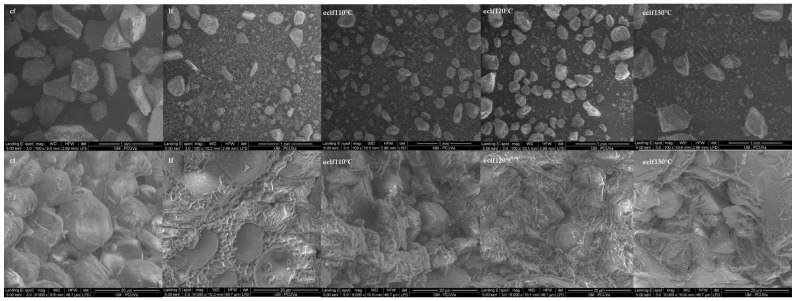
Scanning electron microscope (SEM) of native (corn and lentil) and blend lentil-corn extruded flours at different temperatures. Abbreviations: cf: corn flour; lf: lentil flour; elcf: extruded lentil (50%)–corn flour.

Scanning electron microscopy (SEM) images were obtained to evaluate the effect of extrusion on the microstructure of the different flour samples (Figure 5). Native flours, corn and lentil, showed typical starch granule morphologies. In the case of corn, the structure of the granules showed polyhedric faces and irregular shapes, with sizes in the range of 5 to 20 µm, as previously described [85]. Lentil starch granules showed smooth surfaces, with spherical and kidney-like shapes, and sizes of 10 to 20 µm, results in accordance with Jane et al. [85]. The starch granules appeared surrounded by other cellular and extracellular material, corresponding to a complex matrix mostly composed of protein and fibre fractions [86].

In the case of lentil, the non-starch fraction was more evident than in the case of corn, where the majority of the volume was occupied by the starch granules. The blend samples (lentil 50%) extruded at different temperatures showed modifications in the microstructure of their starch granules. After extrusion, protein and starch interactions occur [87], which may explain the observed loss of the smooth surface of the starch granules, as compared to those of native flours. The increase in temperature produced a higher appearance of irregular extracellular material surrounding the starch granules, as it is more evident in the flours extruded at 130 °C, as compared to those processed at 110 and 120 °C. The material surrounding starch granules may consist of denatured proteins with relatively higher hydrophobicity and reduced solubility [88], which may result in impaired access of amylase to the starch granules, an effect that may explain the lower GI values observed in samples extruded at higher temperatures (130 °C).

### 2.2. Optimization of Cooking Extrusion Concentration Pulse for Blend Mixture (Step 2)

**Figure 6:**
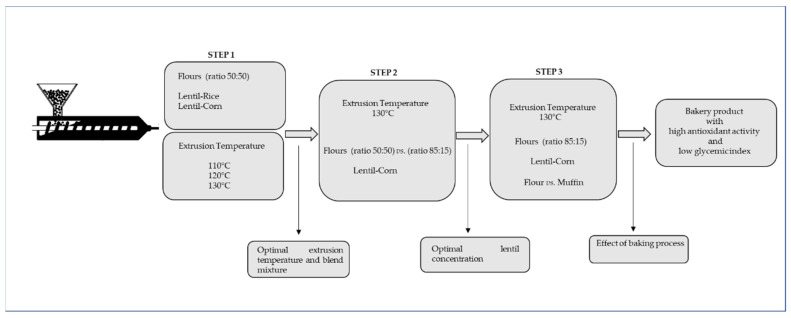
Experimental design.

The effect of pulse concentration of the flour mixture was also investigated. Samples with two different lentil concentrations (15% and 50%) were mixed with optimal cereal (corn) and extruded at optimal temperature (130 °C), as obtained in step 1 (Figure 6). Proximal composition results (Table 4) showed that moisture content was double in 50% lentil sample than 15%. This may respond to the differences in fibre content of the blend flours (4.20 vs. 8.20 g 100 g^−1^ fibre in 15% vs. 50% lentil samples) and the interaction of the cellulose hydroxyl groups with water molecules, resulting in higher water retention [89]. Samples with 50% lentil showed increased protein concentration (15.82 g 100 g^−1^) as compared to 15% lentil (9.13 g 100 g^−1^), values as expected from the protein content of native flour (Table 1). Protein hydrophobicity increases due to denaturation after extrusion process and this may cause a higher interaction of protein and fat, which may correspond to the lower fat values observed in 50% lentil extruded blend, a sample which showed higher protein content than 15% lentil. Phytic acid content more than doubled in the 50% lentil sample, reaching 0.23 g 100 g^−1^, compared to 0.09 g 100 g^−1^ in the 15% sample.

Effect of the lentil concentration was observed in colour parameter changes (Supplementary Table S2). Higher lentil content (50%) significantly reduced luminosity (L*) and b* of the samples, as compared to those containing 15% lentil, with no changes in the a* parameter. Luminosity of the 15% extruded blend (76.39) was in the range of the native flours (72.52 and 74.23, corn and lentil respectively), but an important reduction to 46.30 L* value was obtained in the 50% lentil extruded sample. This effect may respond to the higher protein content of the 50% sample, which will favour Maillard reactions and melanoidin production, with the subsequent darkening effect. Conversely, the decrease of b* value could be explained by the higher b* values of corn native flour, as a reflection of the carotenoid content [90].

The total phenolic content of the 15% and 50% lentil extruded blends increased with increased lentil content (Table 5). As observed from step 1 results (Figure 4I), lentil TP content was nearly six times that of the corn native flour.

The observed result in TP values of extruded 15% lentil was more than half that of the 50% lentil sample (60 and 110 mg GAE 100 g^−1^ respectively), higher than may be expected from native flour contribution. This effect of higher phenolic compound solubilisation efficiency may be due to improved gelatinisation of starch in samples containing lower protein values, as is the case of 15% lentil when compared to the 50%. In the case of ABTS•+ (Figure 2IV) and Q-ABTS•+ results (Table 4), the extruded sample with 15% lentil concentration followed a similar trend to that of TP results and showed higher values than expected, approximately 60% and 90% of that measured in the sample extruded with a 50% of lentil flour, for ABTS•+ and Q-ABTS•+, respectively. The other antioxidant markers showed differences between the 15% and 50% lentil samples, which can be explained by the contribution of each of the native flours (lentil and corn), as evaluated in step 1 (Figure 2II,III,V).

A principal components analysis (Supplementary Figure S2) was performed in order to obtain an overview of the similarities and differences between extruded samples with 15 and 50% lentil concentration and the native corn and lentil flours. The first component (PC1) explained 92.23% and the second (PC2) explained 5.33% of the total variance of the data. The analysis showed that lentil concentration produced a separation of samples. The differences between flours extruded with 15% or 50% lentil content were better explained in base of FRAP results. The increase of lentil flour in the feeding mixture of the extruder improved the reducing power of the obtained flour. Conversely, certain antioxidant markers (ORAC, DPPH, Q-DPPH) explained differences between 15% and 50% lentil flours, to a lower degree than FRAP. Results of ABTS•+, TP and Q-ABTS•+ antioxidant assays did not change significantly with increasing concentration of lentil (15 to 50%). Meanwhile FRAP was a response factor that correlated well with the temperature of extrusion (Supplementary Figure S2) the change in lentil concentration also seems to have an important effect on FRAP values, over other antioxidant markers studied.

The starch content and GI of the samples extruded with 15% and 50% lentil were evaluated (Supplementary Figure S3). The starch content was significantly different and higher in the 15% lentil sample, as expected, due to the higher starch contribution from corn flour. The glycemic index of the 15% lentil sample also showed a higher value (89), as compared to the sample with a 50% lentil (79). Nevertheless, the values obtained for these two samples were maintained lower than those observed in step 1 for flours extruded at 110 and 120 °C (90–100), highlighting the significance of the extrusion temperature in this parameter.

### 2.3. Effect of Baking Process (Step 3)

Based on results obtained from Step 2, a 15% lentil flour extruded at 130 °C was selected for evaluation of the baking process. Moreover, previous studies have considered amounts of legumes not exceeding 30% to improve the amino acid composition, while keeping the sensory properties (hardness, crispiness, lightness) sufficiently similar to controls. Levels of addition up to 15% were previously found to be optimal for Patil et al. [24]. The extruded flour was formulated in a baked product and the effect of thermal processing on the antioxidant and glycemic index properties was evaluated, with the aim to assess the stability of the optimised properties after the baking process.

The results showed that the baked product suffered significant reductions of TP and most of the antioxidant markers evaluated (Table 6). The reducing power (FRAP) was the only antioxidant marker that maintained similar values after baking to those of the extruded flour. The loss of antioxidant properties after a high temperature processing such as baking has been previously reported. Blanch and Ruiz del Castillo [91] found significant TP loss after baking in a corn matrix. Oboh et al. [92] also found significant reduction after roasting of the antioxidant parameters (TP and DPPH) in two maize varieties but observed a different trend in the FRAP results, attributing this potentially to the formation of Maillard compounds. The levels of antioxidant capacity of the final baked product obtained were similar to those of the initial native flours.

The baking process increased the GI of flours; however, the extrusion helped to maintain initial GI values after baking (89 and 94, flour and muffin, respectively; data not shown).

## 3. Materials and Methods

### 3.1. Chemicals

2,2′-Azinobis 3-ethylbenzothiazoline-6-sulfonic acid (ABTS•+), 2,2′-diazobis-(2-aminodinopropane)-dihydrochloride (AAPH), fluorescein, 2,2-diphenyl-1-picrylhydrazyl (DPPH), Folin–Ciocalteu (FC) reagent, gallic acid (GA), and 6-hydroxy-2,5,7,8-tetramethyl-2-carboxylic acid (Trolox) were obtained from Sigma-Aldrich, Co. (St. Louis, MO, USA).

### 3.2. Raw Material

Lentil (*Lens culinaris*), corn (*Zea mays*) and rice (*Oryza sativa*) flours (lf, cf and rf, respectively) were used in this study. All the flours were kindly supplied by a local milling company (Molendum, Zamora, Spain) and Emilio Esteban (Renedo de Esgueva, Valladolid, Spain).

### 3.3. Experimental Design

A multistep sequential design (Figure 1) was studied in order to select the conditions for the development of an antioxidant and low glycemic gluten-free backed product. In a *first step,* since extrusion temperature has a large influence on the properties of the extrudate, overall, in the starch and bioaccessibility of phenolic compounds bounded to fibre, barrel temperatures at exit section of 110 °C, 120 °C, and 130 °C were evaluated. In addition, the effect of two different cereal used in the formulation was studied, extruding 100% rice (erf) and 100% corn (ecf) flours and lentil (50%) with corn (elcf50%) or rice flour (elrf50%). A *second step* was conducted using optimal conditions obtained in step 1, i.e., 130 °C temperature and corn as blend cereal. Two lentil:corn blends were compared, 15:85 (130 °Celcf15%) vs. 50:50 (130 °Celcf50%). Finally, in a *third step*, extruded flour with optimal conditions according to step 1 and 2 outcomes, 130 °C temperature and 15% lentil (130 °Celcf15%), was formulated in a baked gluten-free product (130 °Celcm15%) in order to assess the stability of the antioxidant capacity and glycemic index in a final product.

### 3.4. Extrusion Process

Native (non-extruded), lentil-corn and lentil-rice blend flours, were prepared both in a ratio of 50:50 or 15:85 according to the design (Figure 6). The moisture content of the final mixture was adjusted to 200 g kg^−1^ and maintained overnight at room temperature to favour homogenous flour hydration. Moisture content was determined with a moisture analyser (Sartorius mod. MA35, GmbH & Co. KG, Otto-Brenner-Strasse, Germany).

Corn, rice and blend flours were extruded using a single-screw lab-scale extruder (Brabender mod.KE19 20 DN, Duisburg, Germany). The screw was 19 mm in diameter with a length to diameter ratio of 25 (L/D). The feed rate was fixed at 150 rpm and resulting pressure monitored. The profile temperature of the first three-barrel sections was set at 30, 50 and 90, from Section 1 at the feeding point to Section 3, respectively. The temperature at die/exit Section 4 was set at 110, 120, and 130 °C. The extruded flours were dried overnight at room temperature and after milled using a mill (Model Cyclotec 1093, Foss, Hilleroed, Denmark) fitted with a 0.5 mm screen. Extruded flours were stored in air-tight polyethylene/plastic bags in dark conditions at room temperature until further analyses.

### 3.5. Muffin Product Elaboration

Five muffin doughs were individually prepared by mixing 25 g of the optimal selected extruded mixture flour (step 3) with 22.5 g of sugar, 22.5 g of oil, 23 g of whole egg, 1.5 g of baking powder, and finally 0.5 g NaCl. The doughs were baked using a convection oven at 180 °C for 20 min. The muffins were allowed to cool down at room temperature overnight and freeze-dried in order to stabilize samples during storage. Afterwards, samples were pooled and powdered using a blender (Model M20, IKA-Werke, Staufen im Breisgau, Germany) fitted with a 0.5 mm screen and store in sealed polyethylene/plastic bag at dark conditions to ensure stability until analysis.

### 3.6. Proximal Composition

Moisture content was measured gravimetrically by drying samples at 100 °C for 24 h. Total protein content was determined by the Dumas method, AOAC method 990.03 [93], in an elemental analyser. A conversion factor of 5.7 was used to calculate protein content from nitrogen values. Total fat content was determined using dried samples extracted with petroleum ether (BP 40–60 °C) during 4 h in a Soxtec fat extracting unit (AOAC 2005, method 2003.05) [93]. Ash content was determined by sample incineration in a muffle furnace at 550 °C for 5 h (AOAC 2005, method 923.03) [93]. Carbohydrates were estimated by difference. Total dietary fibre (TDF) content was evaluated using a kit provided by Sigma (TDF100A-1KT, St. Louis, MO, USA), in accordance with manufacturer’s instructions, based on AOAC method 985.29 [93]. Phytic acid, and total phosphorus content were determined using a kit from Megazyme (K-PHYT, Wicklow, Ireland). All parameters were evaluated in duplicate. Proximal composition analysis was expressed in g 100 g^−1^ of fresh matter (FM) with the exception of phytic acid which was expressed g 100 g^−1^ of dry matter (DM).

### 3.7. Colorimetric Analysis and Image Analysis

The colour parameters lightness (L*), redness (a*) and yellowness (b*) were measured using a colorimeter (Colour Quest XE Hunter Lab, Northants, UK). The illuminant was D65 (colour temperature of 6504 K) and the standard observer was 10°. The colorimeter was standardised using a light trap and a white calibration plate. Flours were place in a petri dish and measurements were taken directly. Five measurements were evaluated by duplicated for the different flours and muffin.

### 3.8. Image Analysis (RGB)

Images of the flours were captured using a digital camera (EOS 4000D, Canon, Ohta-Ku, Tokyo, Japan). A photographic stand with white background and controlled lighting conditions was used in order to obtain images with the same brightness and contrast. The stored digital images were analysed for primary colours (red, green and blue; RGB) changes using image analysis software (ImageJ) [94].

### 3.9. Extract Preparation

One gram of ground (<0.5 mm) sample was extracted with 10 mL of methanol: water (1:1, *v*/*v*; acidified to pH = 2 with 0.1M HCl) in a controlled-temperature orbital shaker (250 rpm, 1 h, 25 °C). After centrifugation (2057× *g* 10 min, 25 °C), the supernatant was collected and filtered (Whatman paper n.1). The residue was then re-extracted with 10 mL of methanol. The combined methanol fractions were adjusted to 25 mL with extracting solvent added through the filter. Extract aliquots were stored at −80 °C until further analysis.

### 3.10. Total Phenol Content (TP)

TPs were measured using the Folin-Ciocalteu method as described by Slinkard and Singleton, with modifications [95]. The absorbance was measured at 765 nm with a microplate reader (Fluostar Omega, BMG, Ortenberg, Germany). Results were expressed as mg gallic acid equivalents (GAE) per 100 g of sample (dry basis) using a calibration curve with Gallic acid as standard (9.8–70 µM). Samples were evaluated in duplicate.

### 3.11. Total Antioxidant Capacity (TAC)

TAC was measured on extracts using 2,2-Diphenyl-1-picrylhydrazyl radical (DPPH•), oxygen radical absorbance capacity (ORAC), ferric reducing ability potential (FRAP) and 2,2′-Azinobis-(3-ethylbenzothiazoline-6-sulfonate (ABTS•+) assays. In addition, DPPH• and ABTS•+• modified methods were applied on solid samples without previous extraction (Q-DPPH• and Q-ABTS•+), in order to evaluate the total antioxidant activity of the samples. Samples were evaluated in duplicate.

#### 3.11.1. DPPH• Radical Scavenging Activity and Q-DPPH• Radical Scavenging Activity

The extract-based DPPH• assay was performed as described by Brand-Williams et al. [96], with modifications. A 120 µM DPPH• working solution in pure methanol was prepared. In a 96-well microplate, a volume of 25 µL of extracts was mixed with 100 µL of milliQ water and 125 µL of DPPH working solution. The decay in absorbance at 525 nm was recorded over 30 min with a microplate reader (Fluostar Omega, BMG, Ortenberg, Germany). Different solution of Trolox (7.5–240 µM) were evaluated to perform a calibration curve. Results were expressed as µmol Trolox Eq. 100 g^−1^ sample (dry basis).

The solid sample-based Q-DPPH• method was assayed according to the procedure by Serpen et al. [97], with modifications. Ten milligrams of powdered solid samples (<300 µm) were mixed with 30 mL of DPPH• working solution (60 µM) prepared in methanol. After incubation at 700 rpm for 30 min (Thermomixer Compact, Eppendorf, AG, Hamburg, Germany), samples were centrifuged at 14,000× *g* for 2 min and the absorbance measured at 515 nm. Results were expressed as µmol Trolox Eq. 100 g^−1^ sample.

#### 3.11.2. Oxygen Radical Absorbance Capacity (ORAC)

The procedure was based on a previously reported method by Ou et al. [98], with modifications. Standard curve of Trolox (7.5–240 mM) and samples were diluted in phosphate buffer (10 mM, pH 7.4). Fluorescence was monitored over 150 min with a microplate reader (Fluostar Omega, BMG, Ortenberg, Germany), using 485 nm excitation and 520 nm emission filters. Results were calculated using the areas under the fluorescein decay curves, between the blank and the sample and expressed as µmol Trolox Eq. 100 g^−1^ sample (dry basis).

#### 3.11.3. ABTS•+•+ Radical Cation Scavenging Activity and Q-ABTS•+• Radical Cation Scavenging Activity

ABTS•+• was evaluated following the method first described by Miller and Rice-Evans [99], as modified by Martin-Diana et al. [100]. The absorbance was measured at 730 nm. Results were expressed as µmol Trolox Eq. 100 g^−1^ sample.

The Q-ABTS•+• method described by Serpen et al. [97], as modified in Martin-Diana et al. [100], was used to evaluate the direct antioxidant capacity of samples. Ten mg of sample were mixed with 30 mL ABTS•+• working solution. A volume of 3 mL methanol: water (50:50 *v*:*v*) was added to sample assays to equal the final volume present in the calibration curve run. A calibration curve with Trolox as standard (7.5–240 µM) was used. After incubation for 30 min in darkness, the decay in absorbance was measured at 730 nm. Results were expressed as µmol Trolox Eq. 100 g^−1^ sample (dry basis).

#### 3.11.4. Ferric Reducing Antioxidant Power (FRAP)

FRAP assay was performed following the protocol reported by Pereira et al. [101] Absorbance at 700 nm was recorded. FeSO_4_·7H_2_O was used as the standard (4.0–2.2 mM). The results were expressed as mmol Fe Eq. 100 g^−1^ sample (dry basis).

#### 3.11.5. Relative Antioxidant Capacity (RACI)

RACI was used as an integral concept, which allowed the comparison of antioxidant capacity derived from different chemical methods [101]. RACI values were determined through the following equation: (x − µ)/σ, where x is the antioxidant value, µ is the average value of the results of the corresponding method (TP, ABTS•+, ORAC, DPPH, and FRAP), and σ is the standard deviation.

### 3.12. Glycemic Index (GI)

Before determination of glycemic index (GI), available starch of the samples was first evaluated using a total starch assay kit of Megazyme (K-TSTA 08/16). Afterwards, the in vitro starch hydrolysis rate was determined as described by Gularte and Rosell [102], with slight modifications. The samples containing 50 mg of available starch were dissolved in 2 mL Tris-maleate buffer (0.1 M, pH = 6) and then, 2 mL of enzymatic solution containing porcine pancreatic amylase (460 U/mL) and amyloglucosidase (6.6 U mL^−1^) were added. Aliquots were taken during the incubation period (150 min) and immediately placed in boiling water for 5 min to stop the enzymatic reaction, and then cooled on ice. Then, a volume of 150 µL of absolute ethanol was added and the sample was centrifuged (4 °C, 10.000× *g* for 5 min). The pellet was washed with 150 µL of ethanol:water (1:1, *v/v*) and the supernatants were pooled and stored at 4 °C for the subsequent colorimetric analysis of glucose, using a GOPOD kit (Megazyme, Bray, Ireland). Hydrolysis index (HI) and glycemic index (GI) values were calculated following formulas proposed by Grunfeld [103].

### 3.13. Scanning Electron Microscopy (SEM)

Scanning electron microscopy was conducted using a scanning electronic microscopy (SEM) (FEI QUANTA 200). Samples were cut into square pieces approximately 5 mm long and 5 mm wide. For all samples, images were taken using two standard magnifications, ×100 and ×6000. A voltage of 5 kV was used.

### 3.14. Statistical Analysis

Data were expressed as the mean ± standard deviation. Analysis of variance (ANOVA) and post hoc Duncan’s test was used to identify differences between mean values. Pearson correlation coefficients and principal component analysis (PCA) were performed on centred and standardized data to elucidate the relationships among variables of the phenolic profile and antioxidant capacity of samples. All statistical analyses were performed using Statgraphics Centurion XVI^®^ (StatPoint Technologies, Inc., Warrenton, VA, USA).

## 4. Conclusions

The use of corn in combination with lentil showed improved antioxidant properties and glycemic index as compared to rice. Positive outcomes in the nutritional profile of the extruded flours were observed, such as increased fibre content and reduced antinutritional factor phytic acid. A temperature of 130 °C in the extrusion process enhanced antioxidant properties probably due to the improved of the diffusion of solvent during extraction, enhancing the availability of phenolic compounds. Moreover, a significant reduction of the glycemic index was observed, probably associated to a pastification effect, which limited starch gelatinization during the extrusion processing. As observed from native flour analysis, lentil was expected to provide significantly higher antioxidant properties to the extruded flour blends than corn or rice. A 15% lentil concentration showed similar antioxidant parameters as compared to the 50% lentil extruded flours for most of the antioxidant methods evaluated except for FRAP reducing power test. The antioxidant capacity of the 15% lentil extruded flour muffin resulted in similar levels to those of the initial native flours, and further studies may be of interest in order to support these findings and characterise changes in the antioxidant compound profiles of the native flours and final products.

## Figures and Tables

**Table 1:** Proximal composition (g 100 g^−1^) of native and extruded flours.

PROXIMATE COMPOSITION (g 100 g^−1^ Dry Matter)							
FLOUR	Ash	Fat	Moisture	Protein	Carbohydrates	Fibre	Phytic Acid
**cf**	1.5 ± 0.00 ^b^	0.86 ± 0.02 ^f^	13.64 ± 0.16 ^j^	5.81 ± 0.01 ^a^	78.19 ± 0.13 ^f^	4.05 ± 0.49 ^a^	0.01 ± 0.06 ^abc^
**rf**	0.53 ± 0.00 ^a^	0.86 ± 0.00 ^f^	12.42 ± 0.00 ^i^	7.56 ± 0.00 ^e^	78.63 ± 0.00 ^f^	1.65 ± 0.00 ^a^	0.23 ± 0.01 ^de^
**lf**	2.17 ± 0.01 ^c^	1.42 ± 0.11 ^h^	9.75 ± 0.06 ^c^	24.54 ± 0.13 ^k^	62.13 ± 0.17 ^a^	15.05 ± 2.19 ^c^	0.57 ± 0.29 ^f^
**110°Cecf**	0.28 ± 0.00 ^a^	0.32 ± 0.00 ^d^	11.14 ± 0.00 ^gh^	5.73 ± 0.00 ^a^	82.53 ± 0.00 ^h^	2.10 ± 0.00 ^a^	0.06 ± 0.02 ^ab^
**120°Cecf**	0.32 ± 0.00 ^a^	1.11 ± 0.00 ^g^	10.54 ± 0.00 ^ef^	5.82 ± 0.00 ^a^	82.21 ± 0.00 ^h^	2.17 ± 0.00 ^a^	0.05 ± 0.02 ^a^
**130°Cecf**	1.5 ± 0.00 ^b^	0.50 ± 0.00 ^e^	10.80 ± 0.11 ^efg^	6.20 ± 0.01 ^b^	81.00 ± 0.13 ^g^	2.15 ± 0.35 ^a^	0.08 ± 0.06 ^ab^
**110°Cerf**	0.66 ± 0.02 ^a^	0.10 ± 0.07 ^ab^	15.04 ± 0.25 ^k^	6.88 ± 0.09 ^c^	77.32 ± 0.30 ^e^	22.25 ± 5.89 ^c^	0.30 ± 0.03 ^d^^e^
**120°Cerf**	0.60 ± 0.08 ^a^	0.02 ± 0.01 ^a^	11.01 ± 0.32 ^fgh^	7.10 ± 0.05 ^d^	81.28 ± 0.19 ^d^	20.14 ± 4.96 ^c^	0.28 ± 0.06 ^d^^e^
**130°Cerf**	1.50 ± 0.00 ^b^	0.50 ± 0.00 ^e^	8.89 ± 0.10 ^b^	7.85 ± 0.05 ^f^	81.27 ± 0.05 ^g^	19.0 ± 0.00 ^c^	0.26 ± 0.10 ^d^^e^
**110°Celcf50%**	1.28 ± 0.00 ^b^	0.16 ± 0.00 ^bc^	11.44 ± 0.00 ^h^	15.19 ± 0.00 ^g^	71.93 ± 0.00 ^c^	22.70 ± 0.00 ^c^	0.23 ± 0.02 ^cd^^e^
**120°Celcf50%**	1.31 ± 0.00 ^b^	0.29 ± 0.00 ^cd^	11.40 ± 0.00 ^h^	15.13 ± 0.00 ^g^	71.87 ± 0.00 ^c^	14.91 ± 0.00 ^bc^	0.19 ± 0.02 ^bcd^
**130°Celcf50%**	1.90 ± 0.21 ^c^	0.50 ± 0.00 ^e^	10.06 ± 0.03 ^cd^	15.82 ± 0.09 ^h^	71.73 ± 0.15 ^d^	4.95 ± 4.60 ^a^	0.22 ± 0.02 ^cd^^e^
**110°Celrf50%**	1.43 ± 0.00 ^b^	0.34 ± 0.00 ^d^	11.24 ± 0.00 ^gh^	16.13 ± 0.00 ^i^	70.86 ± 0.00 ^b^	15.87 ± 0.00 ^bc^	0.35 ± 0.05 ^e^
**120°Celrf50%**	1.43 ± 0.00 ^b^	0.38 ± 0.00 ^de^	10.50 ± 0.00 ^d^^e^	16.25 ± 0.00 ^i^	71.44 ± 0.00 ^bc^	15.21 ± 0.00 ^bc^	0.38 ± 0.06 ^e^
**130°Celrf50%**	2.08 ± 0.38 ^c^	0.50 ± 0.00 ^he^	7.15 ± 0.06 ^a^	17.10 ± 0.05 ^j^	73.18 ± 0.38 ^d^	6.75 ± 0.49 ^ab^	0.28 ± 0.10 ^d^^e^
***p*** **-Value**	0.00	0.00	0.00	0.00	0.00	0.00	0.00

Abbreviations: cf: corn flour; rf: rice flour; lf: lentil flour; ecf: extruded corn flour; erf: extruded rice flour; elcf: extruded lentil (50%)-corn flour; elrf: extruded lentil (50%)-rice flour. Different small letters in the same column indicate significant differences (*p* ≤ 0.05) between samples. Ash, fat, moisture, protein, carbohydrate, and fibre are expressed as g 100 g^−1^ of fresh mater basis and phytic acid is expressed as g 100 g^−1^ of dry mater basis.

**Table 2:** Colorimetric parameters (CIE L* a* b*, hue and chroma) of native and extruded flours.

FLOUR	L*	a*	b*	Hue	Chroma
**cf**	72.52 ± 3.69 ^ghi^	8.32 ± 0.46 ^i^	34.04 ± 2.32 ^j^	1.33 ± 0.02 ^a^	35.05 ± 2.28 ^i^
**rf**	84.06 ± 5.06 ^j^	0.52 ± 0.21 ^a^	6.03 ± 0.54 ^a^	1.49 ± 0.03 ^g^	6.05 ± 0.55 ^a^
**lf**	74.23 ± 6.96 ^efg^	0.97 ± 0.30 ^b^	16.23 ± 1.74 ^ef^	1.51 ± 0.02 ^h^	16.27 ± 1.72 ^e^
**110°Cecf**	83.14 ± 4.19 ^j^	3.02 ± 0.48 ^g^	22.62 ± 1.13 ^h^	1.44 ± 0.02 ^e^	22.83 ± 1.17 ^g^
**120°Cecf**	82.43 ± 4.89 ^j^	3.35 ± 0.23 ^h^	25.08 ± 1.31 ^i^	1.44 ± 0.01 ^e^	25.30 ± 1.28 ^h^
**130°Cecf**	52.24 ± 6.22 ^d^	2.75 ± 0.66 ^ef^	17.63 ± 3.83 ^g^	1.42 ± 0.01 ^d^	17.85 ± 3.88 ^f^
**110°Cerf**	73.23 ± 5.56 ^ef^	0.92 ± 0.14 ^b^	7.40 ± 0.31 ^bc^	1.45 ± 0.02 ^ef^	7.46 ± 0.31 ^bc^
**120°Cerf**	82.65 ± 6.20 j	0.65 ± 0.23 ^a^	7.90 ± 0.33 ^bc^	1.49 ± 0.03 ^g^	7.93 ± 0.33 ^bc^
**130°Cerf**	32.64 ± 1.52 ^b^	1.03 ± 0.09 ^b^	8.51 ± 0.53 ^c^	1.45 ± 0.01 ^f^	8.57 ± 0.54 ^c^
**110°Celcf50%**	78.43 ± 1.34 ^i^	2.76 ± 0.14 ^ef^	16.23 ± 0.82 ^e^	1.40 ± 0.01 ^c^	16.46 ± 0.82 ^e^
**120°Celcf50%**	77.87 ± 1.21 ^hi^	2.92 ± 0.15 ^fg^	17.38 ± 0.69 ^d^	1.40 ± 0.01 ^c^	17.63 ± 0.69 ^f^
**130°Celcf50%**	41.70 ± 9.51 ^c^	2.23 ± 0.61 ^hd^	13.55 ± 4.07 ^d^	1.41 ± 0.02 ^cd^	13.73 ± 4.10 ^d^
**110°Celrf50%**	70.87 ± 7.10 ^e^	2.62 ± 0.17 ^e^	13.16 ± 0.92 ^d^	1.37 ± 0.02 ^b^	13.42 ± 0.91 ^d^
**120°Celrf50%**	76.85 ± 2.85 ^hi^	2.36 ± 0.13 ^d^	13.67 ± 0.44 ^d^	1.40 ± 0.01 ^c^	13.87 ± 0.44 ^d^
**130°Celrf50%**	26.83 ± 1.21 ^a^	1.35 ± 0.34 ^c^	6.84 ± 1.10 ^ab^	1.38 ± 0.02 ^b^	6.97 ± 1.14 ^ab^
***p*** **-Value**	0.00	0.00	0.00	0.00	0.00

Abbreviations: cf: corn flour; rf: rice flour; lf: lentil flour; ecf: extruded corn flour; erf: extruded rice flour; elcf: extruded lentil (50%)-corn flour; elrf: extruded lentil (50%)-rice flour. Different small letters in the same column indicate significant differences (*p* ≤ 0.05) between samples.

**Table 3:** Direct antioxidant properties Q-DPPH and Q-ABTS•+ (µmol Eq. Trolox 100 g^−1^ dry matter) of native and extruded flours.

	Direct Antioxidant Properties (Direct Method)	
FLOUR	Q-DPPH (µmol Eq. Trolox 100 g^−1^ Dry Matter)	Q-ABTS•+ (µmol Eq. Trolox 100 g^−1^ Dry Matter)
**cf**	489.40 ± 153.80 ^e^	1340.86 ± 210.15 ^e^
**rf**	65.51 ± 8.16 ^a^	819.98 ± 138.39 ^ab^
**lf**	2100.23 ± 23.96 ^i^	18,844.60 ± 45.22 ^k^
**110°Cecf**	104.39 ± 8.36 ^ab^	1140.03 ± 362.53 ^de^
**120°Cecf**	86.15 ± 8.01 ^ab^	968.35 ± 6.93 ^bcd^
**130°Cecf**	194.28 ± 18.59 ^c^	694.884 ± 42.83 ^a^
**110°Cerf**	38.95 ± 23.62 ^a^	1048.79 ± 136.88 ^cd^
**120°Cerf**	52.73 ± 3.55 ^ab^	893.36 ± 65.19 ^abc^
**130°Cerf**	160.25 ± 0.93 ^bc^	1364.49 ± 81.78 ^e^
**110°Celcf50%**	832.85 ± 17.52 ^bc^	7543.15 ± 65.32 ^h^
**120°Celcf50%**	860.09 ± 80.91 ^f^	5303.76 ± 13,25 ^g^
**130°Celcf50%**	1279.67 ± 43.64 ^h^	8241.97 ± 124.45 ^i^
**110°Celrf50%**	324.25 ± 23.88 ^d^	8228.12 ± 215.19 ^i^
**120°Celrf50%**	753.99 ± 30.85 ^f^	3754.26 ± 88.50 ^f^
**130°Celrf50%**	959.12 ± 71.57 ^g^	9876.41 ± 65.90 ^j^
***p*** **-Value**	0.00	0.00

Abbreviations: cf: corn flour; rf: rice flour; lf: lentil flour; ecf: extruded corn flour; erf: extruded rice flour; elcf: extruded lentil (50%)-corn flour; elrf: extruded lentil (50%)-rice flour. Different small letters in the same column indicate significant differences (*p* ≤ 0.05) between samples.

**Table 4:** Proximal composition (g 100 g^−1^) of flours extruded at 130 °C.

PROXIMATE COMPOSITION (g 100 g^−1^)			
	130°Celcf15%	130°Celcf50%	*p*-Value
**Moisture**	5.00 ± 0.00 ^a^	10.06 ± 0.03 ^b^	0.00
**Protein**	9.13 ± 0.00 ^a^	15.82 ± 0.09 ^b^	0.00
**Fat**	1.29 ± 0.14 ^b^	0.5 ± 0.00 ^a^	0.00
**Carbohydrate**	83.08 ± 0.14 ^b^	71.70 ± 0.14 ^a^	0.00
**Ash**	1.50 ± 0.00 ^a^	1.90 ± 0.21 ^b^	0.01
**Fibre**	4.20 ± 0.00 ^a^	8.20 ± 0.00 ^b^	0.05
Phytic acid *	0.09 ± 0.00 ^a^	0.23 ± 0.03 ^b^	0.00

Abbreviations: elcf: extruded lentil (15% or 50%)-corn flour. Different small letters in the same row indicate significant differences (*p* ≤ 0.05) between samples. Ash, fat, moisture, protein, carbohydrate, and fibre are expressed as g 100 g^−1^ of fresh mater basis. * phytic acid is expressed as g 100 g^−1^ of dry mater basis.

**Table 5:** Total phenol (TP, mg GAE 100 g^−1^ dry matter), total antioxidant activity as ORAC, ABTS•+, DPPH (µmol Eq. Trolox 100 g^−1^ dry matter), and FRAP (mmol Fe^2+^ 100 g^−1^ dry matter) of flours extruded at 130 °C.

FLOUR	TP (mg GAE 100 g^−1^)	ORAC (µmol Eq. Trolox 100 g^−1^)	FRAP (mmol Fe Reduced 100 g^−1^)	ABTS•+ (µmol Eq. Trolox 100 g^−1^)	DPPH (µmol Eq. T rolox 100 g^−1^)	Q-ABTS•+ (µmol Eq. Trolox 100 g^−1^)	Q-DPPH (µmol Eq. Trolox 100 g^−1^)
**130°Celcf15%**	61.90 ± 2.00 ^a^	1694.66 ± 202.83 ^a^	7.76 ± 0.11 ^a^	3504.22 ± 365.78 ^a^	638.50 ± 33.81 ^a^	7590.42 ± 481.87 ^a^	297.44 ± 3 18 ^a^
**130°Celcf50%**	110.58 ± 3.35 ^b^	5039.33 ± 274.37 ^b^	13.37 ± 2.88 ^b^	2936.35 ± 272.85 ^a^	2455.97 ± 286.92 ^b^	8241.97 ± 124.45 ^b^	1279.67 ± 43.64 ^b^

Abbreviations: elcf: extruded lentil (15% or 50%)-corn flour. Different small letters in the same column indicate significant differences (*p* ≤ 0.05) between samples.

**Table 6:** Total phenol (TP, mg GAE 100 g^−1^ dry matter), total antioxidant activity as ORAC, ABTS•+, DPPH (µmol Eq. Trolox 100 g^−1^ dry matter), and FRAP (mmol Fe^2+^ 100 g^−1^ dry matter).

SAMPLE		
	130°Celcf15%	130°Celcm15%
**TP**	61.90 ± 2.00 ^b^	30.26 ± 1.34 ^a^
**ORAC**	1694.66 ± 202.83 ^b^	637.97 ± 75.72 ^a^
**FRAP**	7.76 ± 0.12 ^a^	6.85 ± 0.89 ^a^
**ABTS**	3504.22 ± 365.78 ^b^	752.67 ± 118.50 ^a^
**DPPH**	638.50 ± 33.81 ^b^	228.48 ± 28.80 ^a^
**Q-ABTS**	7590.42 ± 481.87 ^b^	1113.14 ± 427.53 ^a^

Abbreviations: 130°Celcf15%: 15% lentil (balance with corn) flour extruded at 130 °C; 130°Celcm15%: baked product formulated with elcef130°C15% flour. Different small letters in the same row indicate significant differences (*p* ≤ 0.05) between samples.

## Data Availability

Not applicable.

## Supplementary Materials

The following are available online. Figure S1: Pearson’s correlation analysis. (I) Data set of samples: native corn, rice and lentil flours, and extruded corn, rice, lentil (50%)-corn and lentil (50%)-rice flours. (II) Data set of samples: 130°C-extruded lentil (15%)-corn and lentil (50%)-corn flours. (III) Data set of samples: 130°C-extruded lentil (15%)-corn flour and baked product, Figure S2: PCA analysis for native and 130°C-extruded flours. Abbreviations: cf: corn flour; lf: lentil flour; 130°Celcf15%: extruded lentil (15%)-corn flour; 130°Celcf50%: extruded lentil (50%)-corn flour, Figure S3. Starch content (g 100g-1 of dry matter) (I) and Glycemic index (GI) of 130°C-extruded flours (II). Abbreviations: cf: corn flour; lf: lentil flour; 130°Celcf15%: extruded lentil (15%)-corn flour; 130°Celcf50%: extruded lentil (50%)-corn flour. Different small letters indicate significant differences (p ≤ 0.05) between samples, Table S1. RACI of native and extruded flours. Abbreviations: cf: corn flour; rf: rice flour; lf: lentil flour; ecf: extruded corn flour; erf: extruded rice flour; elcf: extruded lentil-corn flour; elrf: extruded lentil-rice flour, Table S2: Colorimetric parameters (CIE L* a* b*, Hue and Chroma) of lentil (balanced with corn) flour extruded at 130°C.

## Sample Availability

The samples are not available from the authors.
