# Supplementary material for: Pulse-Cereal Blend Extrusion for Improving the Antioxidant Properties of a Gluten-Free Flour
Source: Molecules. 2021 Sep 14;26(18):5578. doi: 10.3390/molecules26185578 (PMC8467424; doi:10.3390/molecules26185578)
Supplement: Supplementary file 1 [file molecules-26-05578-s001.zip › molecules-1312246-supplementary.pdf]

Supplementary Materials

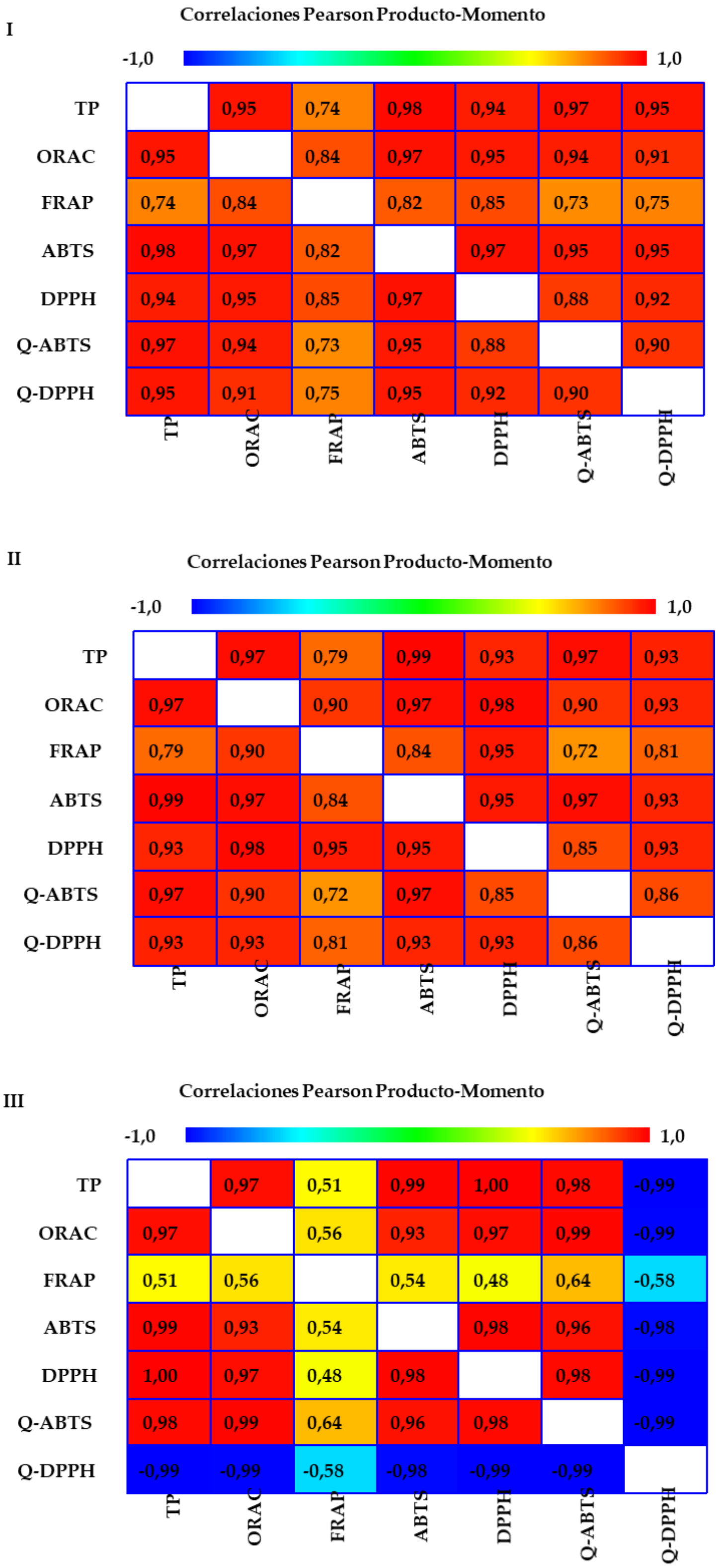

Supplementary Figure S1. Pearson's correlation analysis. (**I**) Data set of samples: native corn, rice and lentil flours, and extruded corn, rice, lentil (50%)-corn and lentil (50%)-rice flours. (**II**) Data set of samples: 130°C-extruded lentil (15%)-corn and lentil (50%)-corn flours. (**III**) Data set of samples: 130°C-extruded lentil (15%)-corn flour and baked product.

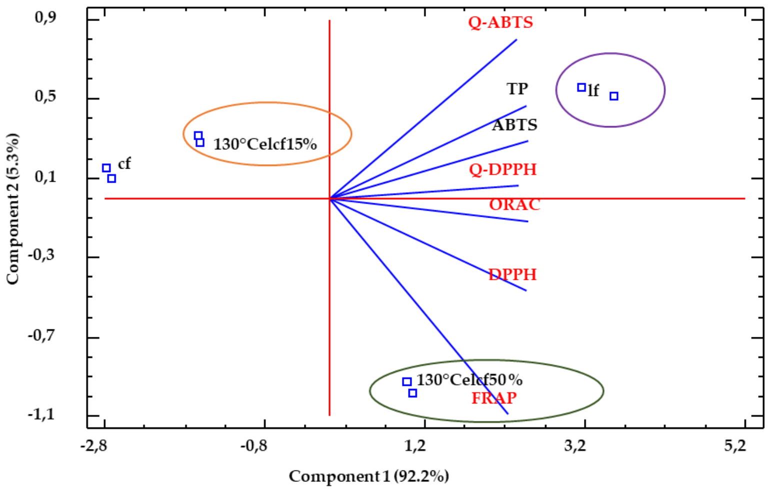

Supplementary Figure S2. PCA analysis for native and 130 °C-extruded flours. Abbreviations: cf: corn flour; lf: lentil flour; 130°Celcf15%: extruded lentil (15%)-corn flour; 130°Celcf50%: extruded lentil (50%)-corn flour.

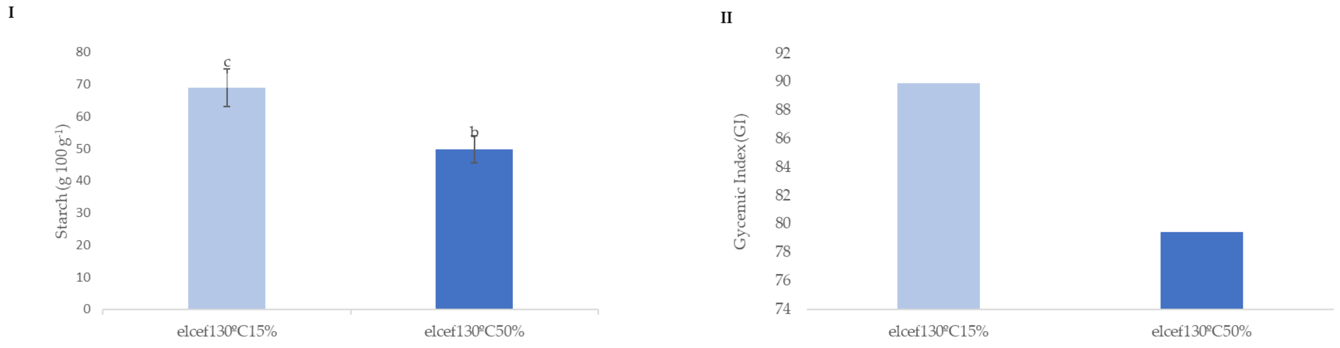

Supplementary Figure S3. Starch content (g 100g^{-1} of dry matter) (**I**) and Glycemic index (GI) of 130 °C-extruded flours (**II**). Abbreviations: cf: corn flour; lf: lentil flour; 130°Celcf15%: extruded lentil (15%)-corn flour; 130°Celcf50%: extruded lentil (50%)-corn flour. Different small letters indicate significant differences ($p \leq 0$ .05) between samples.

Supplementary Table S1. RACI of native and extruded flours. Abbreviations: cf: corn flour; rf: rice flour; lf: lentil flour; ecf: extruded corn flour; erf: extruded rice flour; elcf: extruded lentil-corn flour; elrf: extruded lentil-rice flour.

FLOURS	RACI
cf	−0.56864807
lf	2.43125276
rf	−0.8226027
110°Cecf	−0.82883367
120°Cecf	−0.79126188
130°Cecf	−0.55167058
110°Cerf	−0.85429132
120°Cerf	−0.85760928
130°Cerf	−0.6030281
110°Celcf50%	0.15663327
120°Celcf50%	0.04827246
130°Celcf50%	1.25805807
110°Celrf50%	0.00855723
120°Celrf50%	0.08211183
130°Celrf50%	1.0468099
130°Celcf15%	−0.61092303

Supplementary Table S2. Colorimetric parameters (CIE L* a* b*, Hue and Chroma) of lentil (balanced with corn) flour extruded at 130°C.

Sample	L*	a*	b*	Hue	Chroma
**130°Celcf15%**	76.39 ± 5.27^{b}	3.52 ± 1.21^{a}	21.97 ± 4.10^{b}	1.42 ± 0.04^{a}	22.25 ± 4.22^{b}
**130°Celcf50%**	46.30 ± 14.78^{a}	2.38 ± 0.78^{a}	14.66 ± 5.05^{a}	1.41 ± 0.02^{a}	14.86 ± 5.11^{a}

*Abbreviations: 130°Celcf15%: 15% lentil, 130°Celcf50%: 50% lentil. Different small letters in the same column indicate significant differences ($p \leq 0.05$) between samples.
